# Artificial cerebrospinal fluid use during burr-hole surgery and reoperation rate in patients with chronic subdural hematoma: an analysis using a nationwide inpatient database

**DOI:** 10.1007/s00701-023-05570-1

**Published:** 2023-03-29

**Authors:** Keita Shibahashi, Hiroyuki Ohbe, Hideo Yasunaga

**Affiliations:** 1 Department of Clinical Epidemiology and Health Economics, School of Public Health, The University of Tokyo, 7-3-1 Hongo, Bunkyo-ku, Tokyo, 1130033 Japan; 2 Tertiary Emergency Medical Center, Tokyo Metropolitan Bokutoh Hospital, 4-23-15, Kotobashi, Sumida-ku, Tokyo, 1308575 Japan

**Keywords:** Chronic subdural hematoma, Artificial cerebrospinal fluid, Recurrence, Reoperation, Burr-hole surgery

## Abstract

**Background:**

The optimal surgical procedure to reduce the recurrence rate of chronic subdural hematoma (CSDH) after burr-hole surgery remains to be established. This study aimed to investigate the association between artificial cerebrospinal fluid (ACF) use during burr-hole surgery and reoperation rate in patients with CSDH.

**Method:**

In this retrospective cohort study, we used the Japanese Diagnostic Procedure Combination inpatient database. We identified patients aged 40–90 years who were hospitalized for CSDH and had undergone burr-hole surgery within 2 days of admission, between July 1, 2010 and March 31, 2019. We performed a one-to-one propensity score-matched analysis to compare the outcomes between patients with and without ACF irrigation during burr-hole surgery. The primary outcome was reoperation within 1 year of surgery. The secondary outcome was the total hospitalization costs.

**Results:**

Of the 149,543 patients with CSDH from 1100 hospitals, ACF was used in 32,748 patients (21.9%). Propensity score matching created highly balanced 13,894 matched pairs. In the matched patients, the reoperation rate was significantly lower in the ACF users than that in the non-users group (6.3% vs. 7.0%, *P* = 0.015), with a risk difference of −0.8% (95% confidence interval, −1.5 to −0.2). There was no significant difference in the total hospitalization costs between the two groups (5079 vs. 5042 US dollars, *P* = 0.330).

**Conclusions:**

ACF use during burr-hole surgery may be associated with lower reoperation rate in patients with CSDH.

## Introduction

Chronic subdural hematoma (CSDH) is an old blood collection between the brain surface and dura mater. CSDH is one of the most common neurosurgical conditions encountered. Its incidence is higher in the older population and is rising as the older population increases [12]. The standard treatment for CSDH is a burr-hole surgery. The outcome after burr-hole surgery is generally favorable; however, recurrence rates reportedly range from 4 to 27% [7, 9, 10, 12, 19].

Artificial cerebrospinal fluid (ACF) is an irrigation fluid with a composition akin to that of the human cerebrospinal fluid. Since ACF became commercially available in Japan, they have been used in more than 1000 facilities [1]. ACF is reported to have a hemostatic effect and to minimize cerebrovascular permeability [6, 8]; therefore, the use of ACF as an irrigation solution during burr-hole surgery may improve outcomes in patients with CSDH.

Historical observational studies have reported an association between ACF use during burr-hole surgery and lower recurrence rate in patients with CSDH [1, 9, 21, 22]. However, these were small, single-center studies with insufficient adjustments for confounding factors [1, 9, 21, 22]. Recently, a multicenter randomized controlled trial compared ACF with normal saline as an irrigation solution during burr-hole surgery and found no significant difference in recurrence rate between the two groups [23]. This randomized controlled trial had several limitations [23]. First, it might have been underpowered because of the small sample size. Second, 12% of the participants were lost to follow-up, resulting in an attribution bias. Third, the generalizability of the results is limited because all participating institutions in the study were university hospitals [2]. CSDH is usually treated at general local hospitals, and patients with CSDH treated at university hospitals may differ from those treated at general local hospitals.

To overcome these limitations in the antecedent studies, we aimed to investigate the association between ACF use during burr-hole surgery and the need for reoperation in patients with CSDH, by using a large national database.

## Methods

This cohort study used the Japanese Diagnosis Procedure Combination (DPC) inpatient database. The study protocol was approved by the Institutional Review Board of the University of Tokyo (approval number: 3501-3; December 25, 2017). The requirement of informed consent from the patients was waived due to the retrospective nature of this observational study that analyzed de-identified data.

### Data source

The DPC database is a nationwide database, which includes administrative claims and discharge abstracts from more than 1200 acute care hospitals. It contains the following patient information: hospital type (teaching or non-teaching), age, sex, body weight, height, smoking history (non-smoker, current/past smoker, unknown), activity of daily living scores and level of consciousness at admission, diagnosis (primary diagnosis and comorbidities at admission), drugs used and surgical procedures performed, total hospitalization cost, and discharge status. The International Classification of Diseases, 10th revision (ICD-10), and Japanese medical procedure codes were used to allocate codes for diagnoses and medical procedures, respectively. This database has been validated previously; the specificity of the diagnoses exceeded 96%, the sensitivity of the diagnoses ranged from 50 to 80%, and both the specificity and sensitivity of procedures exceeded 90% [26]. To obtain information about hospitals (university hospitals or not), we linked the database with the facility information and statistical data from the Survey of Medical Institutions 2016 [13].

### Study population

Patients aged 40–90 years who were hospitalized with a primary diagnosis of CSDH (ICD-10 codes: I620, S650) and had undergone first burr-hole surgery for CSDH (Japanese procedure code: K164-2) within 2 days of admission were identified from the database from July 1, 2010 to March 31, 2019. Eligible patients were followed-up for 1 year after the first burr-hole surgery at the same hospital where the surgery was performed.

Patients who received ACF during the first burr-hole surgery were defined as ACF users, and those who did not were defined as ACF non-users.

### Outcomes and covariates

The primary outcome was reoperation of burr-hole surgery being performed during the same hospitalization or readmission at the same hospital within a year of the first surgery. The secondary study outcomes were the presence of postoperative complications (intracranial hematomas and central nervous system infections) and the total hospitalization cost for both the initial admission and readmission. Postoperative intracranial hematoma was defined as intracranial hematoma (ICD-10 codes: S064, S065, I620, I621, I629) that developed after admission. Postoperative central nervous system infections include bacterial meningitis (ICD-10 code: G00) and brain abscess (ICD-10 codes: G060, G062) that developed after admission. Hospitalization costs, recorded in Japanese yen, were converted to US dollars at a rate of 110 yen to 1 US dollar.

The covariates included the type of hospital (teaching or not, university hospital or not), hospital volume per year, fiscal years and season of admission day, admission on weekends or at night, ambulance use, age, sex, body mass index, activity of daily living that was converted to the Barthel Index, comorbidities on admission, Charlson comorbidity index (CCI), level of consciousness, and duration from admission to burr-hole surgery (1: surgery on admission day or 2: surgery on the following day). The hospital volume was calculated as the average number of patients with CSDH who underwent burr-hole surgery at each hospital per year. Fiscal years were categorized as 2010–2013, 2014–2016, or 2017–2019. Age was categorized as 40–49, 50–59, 60–69, 70–79, and 80–89 years. Body mass index was classified based on the World Health Organization criteria for Asian population as follows: < 18.5 kg/m^2^, 18.5–24.9 kg/m^2^, 25.0–29.9 kg/m^2^, ≥ 30 kg/m^2^ [25], and missing data. The Japan Coma Scale (JCS) was used to evaluate the levels of consciousness. The JCS is composed of four main categories: 0 (alert), 1–3 (delirium), 10–30 (somnolence), and 100–300 (coma), which correlated well with the Glasgow Coma Scale [27]. The CCI was calculated based on a validated coding algorithm [16, 17] and was categorized into 0, 1, 2, and ≥ 3. Activities of daily living based on the Barthel Index were categorized as follows: independent (Index 100), moderate dependency (61–99), severe dependency (0–60), and missing data [14].

### ACF in Japan

ACF became commercially available in Japan in 2008 (ARTCEREB®; Otsuka Pharmaceutical Co. Ltd., Tokushima, Japan). The composition of ACF (pH, 7.3; Na^+^, 145 mEq/L; Cl^−^, 129 mEq/L; Mg^+^, 2.2 mEq/L; Ca^2+^, 2.3 mEq/L; HCO_3_^−^, 23.1 mEq/L; P, 1.1 mmol/L; and glucose 0.61 g/L) is more alike to human cerebrospinal fluid (pH, 7.3; Na^+^, 145.5 mEq/L; Cl^−^, 111.9 mEq/L; Mg^+^, 2.2 mEq/L; Ca^2+^, 2.3 mEq/L; HCO_3_^−^, 23.1 mEq/L; P, 1.1 mmol/L; and glucose 0.61 g/L) than normal saline (pH, 6.3; Na^+^, 154 mEq/L; and Cl^−^, 154 mEq/L) and lactated Ringer solution (pH, 6.0–7.5; Na^+^, 130 mEq/L; Cl^−^, 109 mEq/L; Ca^2+^, 3 mEq/L; and HCO_3_^−^, 28 mEq/L) [9, 22].

### Statistical analysis

We used propensity score matching analysis to compare the outcomes between ACF users and non-users. The propensity score was estimated using a multilevel logistic regression model with all covariates. In the model, hospital was used as a random effect variable to account for the differences in ACF use among hospitals. ACF users were subsequently matched to non-users based on one-to-one nearest neighbor matching, without replacement, within an allowable caliper width of 20% set at the pooled standard deviation of the logit of the propensity score [3]. The standardized mean differences of covariates were evaluated to assess matching performance. An absolute standardized mean difference of ≥ 0.1 was considered a meaningful imbalance. In matched patients, outcomes between the two groups in the cohort were compared using the Mann–Whitney *U* test for numeric variables and the chi-squared test for categorical variables. The risk difference with 95% confidence interval (CI) was estimated using a mixed-effects linear regression model. The number needed to treat (NNT) was calculated as the inverse of the absolute risk reduction.

All statistical analyses were performed using R (version 3.6.3; R Foundation for Statistical Computing, Vienna, Austria). All statistical tests were two-sided. Statistical significance was defined as *P*-value < 0.05 or assessed with the 95% CI.

## Results

**Fig. 1:**
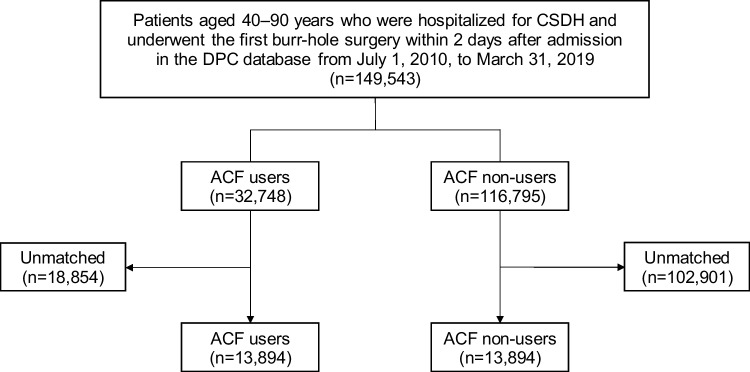
Study flow chart. CSDH, chronic subdural hematoma; ACF, artificial cerebrospinal fluid

**Fig. 2:**
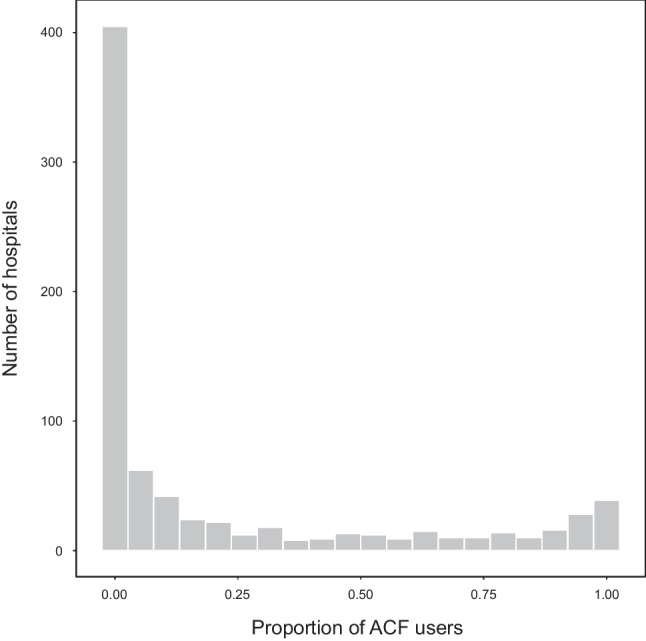
Number of hospitals according to the proportion of artificial cerebrospinal fluid users. ACF, artificial cerebrospinal fluid

We identified 149,543 patients from 1100 hospitals who were admitted for CSDH and had undergone the first burr-hole surgery within 2 days of admission (Fig. 1). Of these, 32,748 (21.9%) patients underwent burr-hole surgery using ACF, and 8797 (5.9%) required reoperation within a year after surgery. The distribution of the proportion of ACF users among the hospitals is shown in Fig. 2.

Table 1 summarizes the baseline characteristics of the two groups before and after propensity score matching. Before matching, ACF was more likely to be used in teaching hospitals or lower-volume hospitals. The proportion of surgical sites was imbalanced between the two groups. Propensity score matching selected 13,894 ACF users and 13,894 non-users. After matching, the baseline characteristics were finely balanced between the two groups, with an absolute standardized mean difference of <0.1. In matched patients, the median volume of ACF used during the first burr-hole surgery was 500 mL (interquartile range, 500–1000 mL).

**Table 1:** Baseline patient characteristics before and after propensity score matching

		Before propensity score matching			After propensity score matching		
		ACF user (*n* = 32,748)	ACF non-user (*n* = 116,795)	ASD	ACF user (*n* = 13,894)	ACF non-user (*n* = 13,894)	ASD
University hospital, *n* (%)		2038 (6.2)	8274 (7.1)	0.035	1529 (11.0)	1559 (11.2)	0.007
Teaching hospital, *n* (%)		27,895 (85.2)	94,247 (80.7)	0.119	11,826 (85.1)	12,076 (86.9)	0.052
Hospital volume per year, mean (SD)		29.9 (15.1)	32.8 (19.1)	0.172	30.2 (15.0)	29.7 (14.5)	0.035
Fiscal year, *n* (%)				0.080			0.044
2010–2012		8645 (26.4)	34,000 (29.1)		4306 (31.0)	4521 (32.5)	
2013–2015		12,218 (37.3)	39,410 (33.7)		4721 (34.0)	4776 (34.4)	
2016–2019		11,885 (36.3)	43,385 (37.1)		4867 (35.0)	4597 (33.1)	
Season, *n* (%)				0.020			0.019
Spring	(Mar–May)	8770 (26.8)	30,773 (26.3)		3737 (26.9)	3634 (26.2)	
Summer	(Jun–Aug)	8122 (24.8)	29,468 (25.2)		3480 (25.0)	3483 (25.1)	
Autumn	(Sep–Nov)	7676 (23.4)	28,038 (24.0)		3204 (23.1)	3290 (23.7)	
Winter	(Dec–Feb)	8180 (25.0)	28,516 (24.4)		3473 (25.0)	3487 (25.1)	
Admission on weekend or at night, *n* (%)		1018 (3.1)	3623 (3.1)	<0.001	403 (2.9)	383 (2.8)	0.009
Ambulance use, *n* (%)		9919 (30.3)	37,268 (31.9)	0.035	4159 (29.9)	4307 (31.0)	0.023
Age, years, mean				0.023			0.014
40–49		497 (1.5)	1929 (1.7)		219 (1.6)	206 (1.5)	
50–59		1419 (4.3)	5113 (4.4)		632 (4.5)	606 (4.4)	
60–69		5112 (15.6)	18,275 (15.6)		2204 (15.9)	2179 (15.7)	
70–79		11,163 (34.1)	40,746 (34.9)		4823 (34.7)	4844 (34.9)	
80–89		14,557 (44.5)	50,732 (43.4)		6016 (43.3)	6059 (43.6)	
Men, *n* (%)		22,517 (68.8)	80,520 (68.9)	0.004	9594 (69.1)	9562 (68.8)	0.005
Body mass index at admission, kg/m^2^, *n* (%)				0.019			0.015
< 16.5		1081 (3.3)	4005 (3.4)		440 (3.2)	460 (3.3)	
16.5–18.4		2861 (8.7)	10,519 (9.0)		1188 (8.6)	1194 (8.6)	
18.5–22.9		12,704 (38.8)	45,109 (38.6)		5340 (38.4)	5399 (38.9)	
23.0–24.9		2827 (8.6)	9643 (8.3)		1229 (8.8)	1196 (8.6)	
≥ 25.0		980 (3.0)	3379 (2.9)		417 (3.0)	417 (3.0)	
Missing		12,295 (37.5)	44,140 (37.8)		5280 (38.0)	5228 (37.6)	
Smoking history, *n* (%)				0.029			0.006
Nonsmoker		19,536 (59.7)	71,327 (61.1)		8231 (59.2)	8272 (59.5)	
Current/past smoker		8483 (25.9)	29,012 (24.8)		3524 (25.4)	3502 (25.2)	
Unknown		4729 (14.4)	16,456 (14.1)		2139 (15.4)	2120 (15.3)	
Barthel Index at admission, *n* (%)				0.064			0.020
0–60		16,634 (50.8)	57,587 (49.3)		7113 (51.2)	7088 (51.0)	
61–99		3574 (10.9)	11,698 (10.0)		1518 (10.9)	1453 (10.5)	
100		6967 (21.3)	24,970 (21.4)		3081 (22.2)	3097 (22.3)	
Missing		5573 (17.0)	22,540 (19.3)		2182 (15.7)	2256 (16.2)	
Comorbidities, *n* (%)							
Myocardial infarction		285 (0.9)	1204 (1.0)	0.017	124 (0.9)	114 (0.8)	0.008
Congestive heart failure		1144 (3.5)	3955 (3.4)	0.006	455 (3.3)	503 (3.6)	0.019
Peripheral vascular disease		239 (0.7)	951 (0.8)	0.010	104 (0.7)	100 (0.7)	0.003
Cerebrovascular disease		3024 (9.2)	10,429 (8.9)	0.011	1342 (9.7)	1283 (9.2)	0.015
Dementia		2785 (8.5)	9537 (8.2)	0.012	1161 (8.4)	1152 (8.3)	0.002
Chronic pulmonary disease		820 (2.5)	3039 (2.6)	0.006	341 (2.5)	332 (2.4)	0.004
Rheumatic disease		165 (0.5)	642 (0.5)	0.006	75 (0.5)	75 (0.5)	<0.001
Peptic ulcer disease		1292 (3.9)	5182 (4.4)	0.025	547 (3.9)	563 (4.1)	0.006
Mild liver disease		873 (2.7)	3079 (2.6)	0.002	360 (2.6)	392 (2.8)	0.014
Diabetes		5676 (17.3)	19,564 (16.8)	0.015	2323 (16.7)	2306 (16.6)	0.003
Hemiplegia/paraplegia		2187 (6.7)	7939 (6.8)	0.005	835 (6.0)	939 (6.8)	0.031
Renal disease		602 (1.8)	2101 (1.8)	0.003	240 (1.7)	257 (1.8)	0.009
Malignancy/lymphoma/leukemia		1333 (4.1)	4756 (4.1)	<0.001	585 (4.2)	594 (4.3)	0.003
Moderate or severe liver disease		46 (0.1)	186 (0.2)	0.005	20 (0.1)	20 (0.1)	<0.001
Metastatic solid tumor		92 (0.3)	341 (0.3)	0.002	43 (0.3)	42 (0.3)	0.001
AIDS/HIV		<5 (0.0)	14 (0.0)	0.006	0 (0.0)	0 (0.0)	<0.001
Liver cirrhosis		173 (0.5)	624 (0.5)	0.001	76 (0.5)	71 (0.5)	0.005
Ischemic cerebrovascular disease		906 (2.8)	3031 (2.6)	0.011	391 (2.8)	385 (2.8)	0.003
Deep venous thrombosis		80 (0.2)	298 (0.3)	0.002	36 (0.3)	38 (0.3)	0.003
Pulmonary embolism		19 (0.1)	78 (0.1)	0.004	12 (0.1)	8 (0.1)	0.011
Valvular heart disease		42 (0.1)	164 (0.1)	0.003	24 (0.2)	18 (0.1)	0.011
Atrial fibrillation		233 (0.7)	782 (0.7)	0.005	94 (0.7)	85 (0.6)	0.008
Charlson comorbidity index, *n* (%)				0.020			0.024
0		17,410 (53.2)	62,988 (53.9)		7517 (54.1)	7466 (53.7)	
1		8492 (25.9)	29,327 (25.1)		3566 (25.7)	3527 (25.4)	
2		4433 (13.5)	15,729 (13.5)		1846 (13.3)	1851 (13.3)	
≥3		2413 (7.4)	8751 (7.5)		965 (6.9)	1050 (7.6)	
Japan Coma Scale at admission, *n* (%)				0.038			0.029
0		14,493 (44.3)	50,555 (43.3)		6056 (43.6)	6123 (44.1)	
1		7020 (21.4)	25,137 (21.5)		2961 (21.3)	2941 (21.2)	
2		4966 (15.2)	17,544 (15.0)		2161 (15.6)	2111 (15.2)	
3		3761 (11.5)	14,146 (12.1)		1637 (11.8)	1596 (11.5)	
10		1222 (3.7)	4348 (3.7)		486 (3.5)	536 (3.9)	
20		343 (1.0)	1277 (1.1)		152 (1.1)	142 (1.0)	
30		309 (0.9)	1092 (0.9)		132 (1.0)	131 (0.9)	
100		296 (0.9)	1199 (1.0)		144 (1.0)	144 (1.0)	
200		268 (0.8)	1127 (1.0)		130 (0.9)	125 (0.9)	
300		69 (0.2)	370 (0.3)		35 (0.3)	45 (0.3)	
Missing		<5 (0.0)	0.0 (0.0)		0 (0.0)	0 (0.0)	
Surgical site, *n* (%)				0.102			0.039
Right		8342 (25.5)	32,048 (27.4)		3757 (27.0)	3804 (27.4)	
Left		10,550 (32.2)	40,024 (34.3)		4775 (34.4)	4871 (35.1)	
Bilateral		3474 (10.6)	13,097 (11.2)		1464 (10.5)	1549 (11.1)	
Not specified		10,382 (31.7)	31,626 (27.1)		3898 (28.1)	3670 (26.4)	
Admission-surgery interval, *n* (%)				0.002			0.032
One day (surgery on admission day)		24,739 (75.5	88,337 (75.6)		10,439 (75.1)	10,630 (76.5)	
Two days (surgery on the following day)		8009 (24.5)	28458 (24.4)		3455 (24.9)	3264 (23.5)	

*ACF*, artificial cerebrospinal fluid; *ASD*, absolute standardized mean difference; *SD*, standardized difference; *AIDS*, acquired immunodeficiency syndrome; *HIV*, human immunodeficiency virus

The study outcomes for the matched patients are shown in Table 2. The reoperation rate was significantly lower in the ACF users than that in the non-users (6.3% vs. 7.0%, *P* = 0.015), with a risk difference of −0.8% (95% CI, −1.5 to −0.2). The estimated NNT was 84 (95% CI, 55 to 178). There was no significant difference in the proportions of postoperative intracranial hematomas (1.0% vs. 0.8%, *P* = 0.088) or postoperative central nervous system infections (0.1% vs. 0.1%, *P* = 0.838) and the total hospitalization costs (5079 vs. 5042 US dollars, *P* = 0.330) between the two groups.

**Table 2:** Comparison of study outcomes between the matched patients

Outcomes	ACF user (*n* = 13,894)	ACF non-user (*n* = 13,894)	*P*-value	Risk difference (95% CI)
Reoperation†, *n* (%)	870 (6.3)	972 (7.0)	0.015	−0.8% (−1.5 to −0.2)
Complications, *n* (%)				
Intracranial hematoma, *n* (%)	140 (1.0)	112 (0.8)	0.088	0.2% (0.0 to 0.5)
Central nervous system infection‡, *n* (%)	13 (0.1)	11 (0.1)	0.838	0.0% (0.0 to 0.0)
Hospitalization cost§, USD, median (IQR)	5079 (4087–6649)	5042 (4076–6576)	0.330	105 (−32 to 243)

†Reoperation within a year after the first burr-hole surgery

‡Central nervous system infection included bacterial meningitis and brain abscess

§Total hospitalization costs for both initial admission and readmission

*ACF*, artificial cerebrospinal fluid; *USD*, US dollar; *IQR*, interquartile range; *95% CI*, 95% confidence interval

## Discussion

Our analysis revealed that ACF use during burr-hole surgery was associated with a significantly lower reoperation rate in patients with CSDH, and there was no significant difference in the hospitalization costs between the ACF users and non-users.

Our results were consistent with those of historical observational studies, though the difference in the reoperation rate between the two groups was much smaller than that previously reported [1, 9]. This discrepancy may be partly explained by the higher recurrence rates in previous studies (15.0% and 23.8%) than in our study (7.0%) and the insufficient adjustment for confounders in the previous studies. The use of a nationwide database enabled us to obtain highly balanced matched patients and derive less-biased results. The most important evidence on this issue is a randomized controlled trial that included 177 ACF users and 165 non-users from ten hospitals in Japan. The study reported a non-significant difference in the recurrence rate after burr-hole surgery for CSDH between the two groups (11.9% vs. 10.9%). However, to detect the observed difference in this study with 80% statistical power and a two-sided type I error rate of 0.05, more than 20,000 samples per group would have been required for a randomized controlled trial. Considering this difficulty, we determined that a large observational study with sufficient adjustment for confounding factors would be ideal as evidence on this issue.

Our findings are consistent with evidence from basic research studies. A study using a mouse model reported that the choice of irrigation solution used in neurosurgical procedures affected the extent of bleeding at the injury site, and ACF irrigation significantly reduced bleeding than normal saline [6]. Calcium is essential for blood coagulation and vascular smooth muscle contraction as they play important roles in hemostasis. The previous study hypothesized that calcium ions included in the ACF may have contributed to the reduced bleeding. A study reported that CSDH membranes irrigated with ACF during burr-hole surgery showed less damage than those irrigated with normal saline; thus, the authors presumed that ACF minimized irritation and promoted healing after surgery [1]. These results suggest that ACF may promote hemostasis of the outer hematoma membrane. In addition, ACF irrigation may reduce recurrence by rinsing out inflammatory mediators from the subdural compartment. Excessive inflammation is one of the main etiological origins of CSDH, and the overproduction of inflammatory mediators is associated with hematoma expansion [5].

Compared to other potential modifications of the surgical procedure to reduce the recurrence rate after burr-hole surgery, ACF use has several advantages. First, it is simple and easy to incorporate in the surgery. Surgeons do not need to change their standard procedure, except for changing the irrigation solution to an ACF. Second, ACF has a favorable safety profile. ACF has a composition that is more similar to that of human cerebrospinal fluid than normal saline. Although normal saline is conventionally used as an irrigation fluid, ACF use is theoretically safer. Studies have reported an association between ACF use during neurosurgery and lower risk of adverse events [15, 23]. Third, ACF is available at a relatively low cost. In Japan, 500 mL of ACF, the median dose used in this study, was available at 16.8 USD. Based on the NNT 84 (95% CI, 55 to 178) in this study, the additional cost needed to reduce one reoperation by changing the irrigation solution from normal saline (1.7 USD for 500 mL) to ACF was calculated to be 1268 (95% CI, 831 to 2688) USD. As this additional cost is presumed to be lower than the hospitalization costs for reoperation [18], ACF use may also be justified in terms of health economics. However, no significant difference in total hospitalization cost in the present study was observed. Further research is needed to clarify this issue.

While ARTCEREB® is not available outside Japan, other types of ACF are in use, such as ACSF (R&D Systems, Inc., Minneapolis, MN, USA) and Elliotts B® Solution (Lukare Medical, LLC, Scotch Plains, NJ, USA). Therefore, our results have clinical implication for surgeons worldwide. Given the aforementioned advantages of ACF, our results suggest that the benefits of ACF use during surgery deserve further consideration. Future prospective trials are needed to confirm our findings and to understand the underlying mechanisms.

### Limitations

This study had several limitations. First, it was a retrospective study. We obtained a highly balanced pair cohort; however, biases due to unmeasured confounders were not eliminated. For example, our dataset does not include information on detailed surgical procedures. It is possible that surgeons who used ACF were eager to improve the surgery with active search, and ACF was merely a surrogate indicator of surgical proficiency. Although burr-hole surgery for CSDH is straightforward and surgical techniques to reduce recurrence are not established [11], the difference in surgical techniques may have biased our results. Additionally, information on the temperature [4] and volume [20, 24] of the rinsing solution, which may contribute to recurrence, was not available. Second, causality cannot be directly referred to. Third, the rinsing solution used in the control group was not recorded. Fourth, hospitalizations in one hospital could not be linked to those in another hospital in the database. Based on clinical experience in Japan, we assumed that reoperations were performed at the same hospital, and the likelihood of undergoing reoperation at a different hospital was unrelated to ACF use; however, if these assumptions do not hold, our results could be biased. Finally, our results require external validation in countries with different demographic characteristics and surgical procedures.

## Conclusions

ACF use during burr-hole surgery may be associated with lower risk of reoperation in patients with CSDH. Further studies are warranted to confirm our findings and to understand the underlying mechanisms.
